# The Degeneration Paradox: Severely Degenerated Cervical Nucleus Pulposus Cells Display Enhanced Mechanoplasticity Under Moderate Cyclic Tensile Strain

**DOI:** 10.3390/biom16030461

**Published:** 2026-03-18

**Authors:** Yuwen Wang, Yi Chen, Bowei Xiao, Baining Zhang, Juying Huang, Nan Zhang, Binxuan Wu, Tianhua Rong, Baoge Liu

**Affiliations:** 1 Department of Orthopaedic Surgery, Beijing Tiantan Hospital, Capital Medical University, No. 119 South 4th Ring West Road, Fengtai District, Beijing 100070, China; wangyw1993@mail.ccmu.edu.cn (Y.W.); chenyiiiii@mail.ccmu.edu.cn (Y.C.); mxiao_bowei@163.com (B.X.); zhangbaespine@mail.ccmu.edu.cn (B.Z.); wubxspine@mail.ccmu.edu.cn (B.W.); rongtianhua@mail.ccmu.edu.cn (T.R.); 2 China National Clinical Research Center for Neurological Diseases, Beijing Tiantan Hospital, Capital Medical University, Beijing 100070, China; 3 Beijing Key Laboratory of Fundamental Research on Biomechanics in Clinical Application, Capital Medical University, Beijing 100069, China; huangjy@ccmu.edu.cn (J.H.); zhangnan@ccmu.edu.cn (N.Z.); 4 School of Biomedical Engineering, Capital Medical University, Beijing 100069, China

**Keywords:** cervical intervertebral disc degeneration, nucleus pulposus cells, cell stiffness, F-actin, cyclic tensile strain, mechanotransduction, Degeneration Paradox

## Abstract

Cervical Intervertebral Disc Degeneration (CIVDD) involves significant microenvironmental physical stiffening, forcing nucleus pulposus cells (NPCs) into a rigid phenotype via F-actin over-assembly. It remains unclear if cyclic tensile strain (CTS) can reverse this physical stiffening, particularly in severe degeneration. This study stratified 18 patients into Mild, Moderate, and Severe cohorts based on MRI. Primary NPCs were subjected to physiological 5% CTS (1 Hz, 24 h). Atomic Force Microscopy (AFM) and immunofluorescence were utilized to evaluate Young’s modulus and cytoskeletal remodeling. Results demonstrated that baseline cellular stiffness increased significantly with degeneration severity. Following CTS, all groups exhibited universal de-stiffening and F-actin depolymerization. Crucially, a “Degeneration Paradox” emerged: the Severe group displayed the highest relative elastic modulus recovery rate, significantly surpassing the Mild group. This microscopic recovery correlated inversely with preoperative disc height loss and range of motion. We conclude that severely degenerated cells are not metabolically quiescent but “physically locked” by a rigid cytoskeleton. Physiological CTS restores compliance via mechanical unloading, confirming that severe cells retain superior relative mechanoplasticity and may benefit from mechanotherapy-based “unlocking” strategies.

## 1. Introduction

Cervical Intervertebral Disc Degeneration (CIVDD) serves as the pathological foundation for chronic neck pain and neurological dysfunction. Driven by a significantly younger demographic of onset and the resultant substantial socioeconomic burden, CIVDD has evolved into a pressing global public health challenge [1,2]. The pathophysiological progression involves not only the enzymatic reduction of hydrophilic matrix elements like Type II collagen (Col II) and Aggrecan in the Nucleus Pulposus (NP) but also significant changes in the microenvironment’s physical properties [3,4,5]. Concomitant with desiccation and progressive fibrosis, the NP tissue gradually forfeits its osmotic hydro-absorption capacity and poroelastic buffering properties. Consequently, it transitions from a hydrated, viscoelastic fluid into a desiccated, collapsed, and highly stiffened fibrocartilaginous structure [6,7]. Emerging evidence indicates that this physical stiffening of the extracellular matrix (ECM) is not merely a consequence of degeneration but an instigating driver that precipitates further deterioration of cellular function [8].

Mechanotransduction theory suggests that cells detect the physical stiffness of the external matrix through transmembrane receptors like integrins and adjust to this mechanical setting by reorganizing their cytoskeleton [9,10,11]. Within a stiffened degenerate matrix, to maintain tensional homeostasis, cells hyperactivate the RhoA/ROCK signaling pathway, leading to the pathological assembly and accumulation of F-actin stress fibers [12]. This dense actin network acts as a “physical shackle,” forcing the cell into a distinct “stiffening phenotype” [13,14]. Such physical rigidification not only restricts cellular deformability but may also impede the nucleocytoplasmic shuttling of mechanosensitive transcription factors via steric hindrance effects [15]. This suppression of matrix synthesis at the gene expression level establishes a self-perpetuating vicious cycle of “matrix stiffening–cell stiffening–synthesis inhibition”.

Current clinical non-surgical strategies, such as traction and rehabilitation exercises, are primarily predicated on macroscopic mechanical principles, aiming to mitigate symptoms by altering intradiscal pressure [16]. At the microscopic cellular level, moderate Cyclic Tensile Strain (CTS) has been confirmed to upregulate anabolic activity via mechanochemical signal transduction [17]. However, existing literature has predominantly focused on downstream biochemical pathway alterations, largely overlooking the direct effects of mechanical stimulation on the physical attributes of the cell body itself [18,19,20]. Whether mechanical therapy can serve as a physical key to directly dismantle the excessively accumulated F-actin cytoskeleton and restore juvenile-like compliance remains a significant knowledge gap in the field.

Furthermore, it is imperative to underscore that the restoration of cellular physical compliance is not merely an epiphenomenon of reversed degeneration, but a necessary biomechanical prerequisite for rebuilding the buffering function of NP tissue [21,22]. Similar to osteocytes or chondrocytes, the superior micro-elasticity of NP cells constitutes the primary line of defense against macroscopic spinal loads [23,24,25]. If cells fail to break the internal rigid cytoskeletal network via de-stiffening, they remain vulnerable to focal stress concentration under physiological spinal loading, leading to membrane rupture or organelle damage—even if matrix synthesis genes are transiently upregulated pharmacologically. Therefore, identifying strategies to reset cytoskeletal tension via physical means may represent the critical bridge spanning the translational chasm between gene expression recovery and functional tissue reconstruction.

A more critical clinical scientific inquiry pertains to therapeutic decision-making for patients with severe degeneration. Traditional dogmas posit that in patients presenting with Miyazaki grades IV–V, NP cells have undergone severe senescence, apoptosis, and fibrosis, rendering them functionally exhausted. Consequently, such cases are deemed refractory to conservative management, and surgical intervention is often the immediate recommendation [26]. However, clinical observations frequently reveal that a subset of patients with severe radiographic degeneration experiences significant symptomatic relief following standardized physical therapy. This phenomenon suggests that severely degenerated cells may not be in a state of total metabolic silence but are rather physically locked by a rigid environment and cytoskeleton. Once this physical lock is released, they may retain surprising repair potential.

Predicated on these clinical paradoxes and biomechanical mechanisms, this study proposes a core hypothesis: Cell stiffness is a pivotal biomechanical biomarker reflecting the severity of CIVDD, rooted in the pathological accumulation of F-actin; furthermore, 5% physiological CTS can induce F-actin depolymerization via a “Physical Unloading” mechanism, thereby reversing cell stiffening and restarting anabolism. We further postulate that due to their high baseline tensional state, severely degenerated cells may be uniquely sensitive to this physical relaxation, exhibiting unexpected mechanoplasticity. By utilizing Atomic Force Microscopy (AFM) combined with immunofluorescence, this study systematically evaluates the differential mechanical responses of human cervical NP cells across varying stages of degeneration. Our objective is to elucidate the latent biomechanical recovery value of severely degenerated cells, providing a novel theoretical basis for stratified therapeutic strategies in cervical spondylosis.

## 2. Materials and Methods

### 2.1. Patient Recruitment and Clinical Assessment

The study protocol was approved by the Institutional Ethics Committee (Approval No. KYSQ 2021-322-02). Written informed consent was obtained from all participants prior to surgery.

A total of 18 intervertebral disc (IVD) specimens were harvested from patients undergoing anterior cervical discectomy and fusion (ACDF) for cervical spondylosis between September 2023 and September 2025. The inclusion criteria were as follows: (1) Diagnosed with degenerative cervical radiculopathy and/or cervical myelopathy confirmed by clinical symptoms and physical examination; (2) Aged between 50 and 69 years (to minimize age-related bias); (3) Failure of conservative treatment for at least 3 months. Exclusion criteria encompassed: (1) a history of atlantoaxial deformity or dislocation; (2) cervical instability; (3) a history of cervical trauma; (4) presence of infection, tumor, or tuberculosis; (5) prior history of cranial or spinal surgery; and (6) connective tissue disorders (e.g., rheumatoid arthritis, ankylosing spondylitis) or systemic neuromuscular diseases.

For radiographic assessment, standard lateral radiographs of the cervical spine were obtained in the neutral position using a uniform digital radiography system (GE Discovery XR656; GE Healthcare, Chicago, IL, USA). Functional radiographs were acquired by instructing patients to maximally flex and extend the cervical spine to obtain hyperflexion and hyperextension views, respectively. Additionally, preoperative magnetic resonance imaging (MRI) was performed on all subjects.

Degeneration of the target segment was evaluated by two senior orthopedic surgeons in a double-blinded manner according to the Miyazaki grading system [27]. Briefly, this system evaluates T2-weighted midsagittal images based on four parameters: nucleus signal intensity, nucleus structure, distinction of nucleus and annulus, and disc height. The grades are defined as follows: Grade I (hyperintense, homogeneous white structure, clear distinction, normal height); Grade II (hyperintense, inhomogeneous structure with a horizontal white band, clear distinction, normal height); Grade III (intermediate intensity, inhomogeneous gray to black structure, unclear distinction, normal to decreased height); Grade IV (hypointense, inhomogeneous gray to black structure, lost distinction, normal to decreased height); and Grade V (hypointense, inhomogeneous gray to black structure, lost distinction, collapsed disc space) (Figure S1). In cases of inter-observer discrepancy, a third senior consultant adjudicated the final grade. Consequently, patients were stratified into three cohorts: the Mild group (Miyazaki grades I–II), the Moderate group (Miyazaki grade III), and the Severe group (Miyazaki grades IV–V).

To semi-quantitatively assess the severity of collapse, the percentage of disc height loss was calculated relative to the average Degree of Height Loss of adjacent normal segments. Based on the extent of loss, specimens were assigned a score: 1 point for height loss ≤ 25%; 2 points for loss >25–≤50%; and 3 points for loss >50–≤75%.

Segmental range of motion (ROM) was calculated as the difference in the Cobb angle (specifically defined as the angle formed by the intersection of a line drawn parallel to the inferior endplate of the superior vertebral body and a line parallel to the superior endplate of the inferior vertebral body at the targeted degenerative level) of the target segment between hyperextension and hyperflexion radiographs [28]. Based on ROM values, subjects were categorized into three functional groups: the Stiffness group (0–5°), the Restricted group (6–10°), and the Mobile group (11–15°).

Detailed demographic and baseline clinical characteristics of the study cohort are summarized in Table 1. Furthermore, the core cellular biomechanical variables presented in this table (i.e., Elastic modulus, Post-CTS Elastic modulus, and the Rate of change) were quantitatively evaluated using AFM and the Flexcell tension system; the detailed experimental protocols for these specific measurements are comprehensively described in Section 2.3 and Section 2.4.

### 2.2. Isolation and High-Purity Culture of Primary Nucleus Pulposus Cells (NPCs)

**Figure 1:**
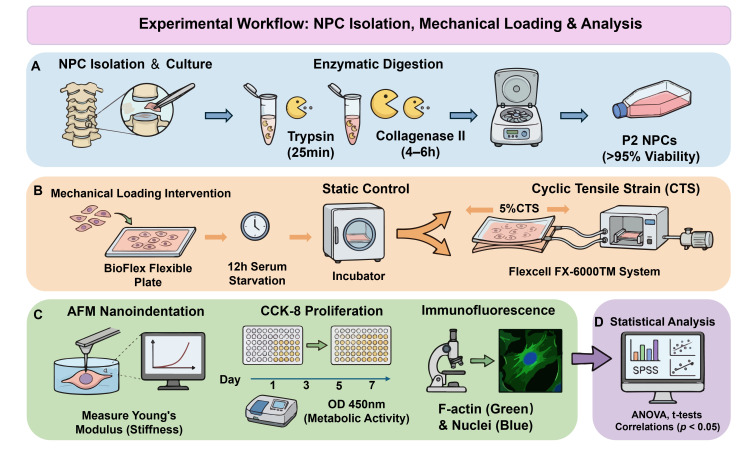
Schematic illustration of the experimental design and workflow. The study involved three main phases: (**A**) Isolation and culture of primary human cervical nucleus pulposus cells (NPCs) up to passage 2 (P2); (**B**) Application of cyclic tensile strain (CTS) intervention (5% elongation, 1 Hz, 24 h) using the Flexcell system, evaluated against their static baseline (Pre-CTS); (**C**) Multi-dimensional analysis including biomechanical testing (AFM), proliferation assay (Cell Counting Kit-8 (CCK-8)), and morphological observation (Immunofluorescence). (**D**) Statistical analyses were performed to correlate cellular responses with clinical parameters.

Intraoperatively, nucleus pulposus (NP) tissue was meticulously excised under microscopic guidance as illustrated in the experimental workflow (Figure 1). To prevent contamination by heterogeneous cell populations, the annulus fibrosus, cartilaginous endplates, and blood clots were rigorously removed. The tissue specimens were washed three times with phosphate-buffered saline (PBS) and minced into fragments of approximately 1 mm^3^.

Cells were isolated using a sequential two-step enzymatic digestion protocol. First, the fragments were digested with 3 volumes of 0.25% trypsin (VivaCell, Shanghai, China) at 37 °C for 25 min. Digestion was neutralized by adding an equal volume of complete medium containing 17% fetal bovine serum (FBS; Vivacell, China), followed by centrifugation at 1200 rpm for 5 min to discard the supernatant. Subsequently, the tissue was digested with 3 volumes of 0.2% collagenase type II (Lablead, Beijing, China) at 37 °C for 4–6 h. The resulting suspension was filtered through a cell strainer (Corning, Corning, NY, USA) and centrifuged at 1200 rpm for 5 min to collect the cells.

The cellular pellet was resuspended in PBS, washed via centrifugation at 1000 rpm, and finally resuspended in Dulbecco’s Modified Eagle Medium/Nutrient Mixture F-12 (DMEM/F-12) (VivaCell, China) supplemented with 15% FBS and 1% penicillin-streptomycin (VivaCell, China). Primary cells were seeded into 25 cm^2^ cell culture flasks (Thermo Fisher Scientific, Waltham, MA, USA) and 6-well plates (Corning, USA) containing 24 mm diameter coverslips (Solarbio, Beijing, China), then maintained at 37 °C in a 5% CO_2_ atmosphere.

An equal volume of complete medium was supplemented 3 days post-seeding. The medium was fully replaced for the first time on day 5 and subsequently refreshed every 2 days. Upon reaching >90% confluence, cells were subcultured at a 1:2 ratio using 0.25% trypsin. To ensure experimental consistency, this study strictly utilized only second-passage (P2) cells with a viability >95%, as confirmed by Trypan blue staining (Solarbio, Beijing, China).

### 2.3. Dynamic Cyclic Tensile Strain Loading System

Second passage (P2) NPCs were seeded at a density of 2 × 10^5^ cells/well into Type I collagen-coated BioFlex^®^ six-well flexible silicone-bottomed culture plates (Flexcell International Corp., Burlington, NC, USA). Upon reaching 70–80% confluence, cells were subjected to serum starvation in serum-free medium for 12 h to synchronize the cell cycle and eliminate the confounding effects of serum-derived growth factors.

Subsequently, mechanical stimulation was applied using the Flexcell FX-6000™ Tension System (Flexcell International Corp., Burlington, NC, USA). This system generates precise radial tensile strain via a computer-controlled vacuum pump that regulates negative pressure beneath the flexible silicone membrane. The loading protocol was designed to mimic the physiological daily activity patterns of the human cervical spine, utilizing the following parameters: 5% elongation, 1 Hz frequency (sinusoidal waveform), and a duration of 24 h.

For baseline (Pre-CTS) evaluations, cells were seeded in identical BioFlex^®^ plates and maintained in the same incubator under static conditions without connection to the vacuum manifold. This setup ensured that any observed differences were due to mechanical strain rather than variations in substrate material or culture environment.

### 2.4. Single-Cell Nanoindentation via AFM

Cellular morphological characteristics and force-distance curves were acquired using a BioScope Resolve AFM system (Bruker, Karlsruhe, Germany), which is optimized for biological specimen analysis in fluid environments.

MLCT-A probes (Bruker, Karlsruhe, Germany) with a nominal spring constant of k = 0.07 N/m were utilized and mounted on a fluid probe holder. Scanning was performed on adherent cells cultured in 35 mm Petri dishes immersed in a physiological fluid environment. Measurements were conducted at room temperature in Force-Volume mode, with a scan rate of 1 Hz and a sampling density of 64 points per scan line to ensure high resolution.

For sampling, five representative short spindle-shaped cells (the predominant morphology of P2 NPCs as characterized in previous studies [29,30]) were randomly selected from each culture plate. Guided by the height sensor for precise localization, nanoindentation testing was performed at ten distinct points randomly distributed within the nuclear region of each cell. To maximize cell viability and ensure data fidelity, the duration of each testing session was strictly limited to 40 min, and any sampling points exhibiting atypical stress relaxation curves were excluded.

Finally, Young’s modulus was calculated by fitting the approach segment of the force curves to the Hertz model using NanoScope Analysis 1.5 software. The calculation formula is as follows:

(1) $$F=\frac{2}{\pi }\frac{E}{1-\nu^{2}}\delta {‌}^{2}\times tan(\alpha )$$

where *F* represents the loading force, α is the half-opening angle of the probe tip,  *ν* denotes the Poisson’s ratio of the cell (assumed to be 0.5 for incompressible biological materials), and *δ* represents the indentation depth.

To strictly correspond with the clinical macroscopic variables presented in Table 1, the specific biomechanical evaluation metrics are defined as follows: The baseline “Elastic modulus” (E_baseline_) was defined as the cell stiffness measured under static control conditions (Pre-CTS). The “Post-CTS Elastic modulus” (E_CTS_) was defined as the stiffness evaluated immediately after the 24-h 5% CTS intervention. Additionally, to quantify the mechanical recovery potential following CTS intervention, the relative rate of change in the elastic modulus (ΔE%) was calculated using the following formula: ΔE% = (E_baseline_ − E_CTS_)/E_baseline_ × 100%.

### 2.5. Cell Proliferation Assay (CCK-8)

To evaluate the functional impact of mechanical stimulation on cellular vitality, the proliferation dynamics of NPCs isolated from all 18 specimens were quantified using the Cell Counting Kit-8 (CCK-8) assay (Dojindo, Tokyo, Japan).

Following the completion of the 24-h CTS protocol, the culture medium was refreshed. Longitudinal assessment of cell proliferation was conducted at days 1, 3, 5, and 7 post-intervention. At each designated time point, the culture medium was supplemented with 10% (*v*/*v*) CCK-8 solution, and cells were incubated at 37 °C for 2 h in the dark to allow for the formation of water-soluble formazan. Subsequently, the optical density (OD) was measured at a wavelength of 450 nm using a microplate spectrophotometer. The OD value served as a surrogate marker for the metabolic activity and proliferative capacity of the cells.

### 2.6. Immunofluorescence Staining

To visualize the cytoskeletal remodeling of NPCs under varying stiffness and intervention conditions, cells from each group were subjected to immunofluorescence staining.

Following two washes with PBS, cells were fixed with 4% paraformaldehyde (PFA) for 30 min and subsequently permeabilized with 0.3% Triton X-100 in PBS for 10 min at room temperature. To prevent non-specific binding, samples were incubated with blocking buffer for 1 h at 4 °C.

F-actin filaments were stained using FITC-conjugated phalloidin (FITC-Phalloidin, CA1260; Solarbio, China), and nuclei were counterstained with DAPI for 30 min.

Immunofluorescence images were acquired using an Axiovert 40CFL fluorescence microscope (Zeiss, Oberkochen, Germany). To ensure the comparability of fluorescence intensity, all images were captured under identical excitation/emission parameters and gain settings. Fluorescence intensity was subsequently measured and quantified using ImageJ software (Version 1.48v; National Institutes of Health, Bethesda, MD, USA). Specifically, background subtraction was performed using the rolling ball algorithm (radius = 50 pixels). The F-actin region of interest (ROI) was defined by the cell boundary, and the nuclear area (DAPI-positive mask) was explicitly excluded to isolate cytoplasmic F-actin fluorescence. For each experimental group, a minimum of 5 random fields of view were captured, and at least 30 individual cells were analyzed to calculate the mean fluorescence intensity (MFI).

### 2.7. Statistical Analysis

Processing protocols for AFM data were identical to those described in Section 2.4. All statistical analyses were performed using SPSS 22.0 software (International Business Machines Corp. (IBM), Armonk, NY, USA). Quantitative data are presented as mean ± standard deviation (SD).

For multi-group comparisons of normally distributed data (e.g., among Mild, Moderate, and Severe groups), Welch’s ANOVA was utilized to account for unequal variances and unbalanced sample sizes, followed by Games-Howell post hoc tests for pairwise comparisons. Comparisons between two independent groups (e.g., Sex and Age subgroups) were performed using the independent Student’s *t*-test, while between baseline and post CTS conditions within the same group stratification were conducted using the paired Student’s *t*-test. For multi-group comparisons of ordinal or non-normally distributed data (e.g., disc height loss score), the non-parametric Kruskal–Wallis test was employed, followed by Dunn’s multiple comparisons test. Non-normally distributed data between two groups were analyzed using the Wilcoxon rank-sum test, while categorical variables were assessed using the Chi-square test.

Furthermore, Pearson correlation analysis was employed for continuous variables (e.g., ROM), whereas Spearman’s rank correlation analysis was strictly utilized to evaluate associations involving ordinal variables (e.g., Miyazaki degeneration grade and the degree of intervertebral disc collapse) and the AFM-derived mechanical recovery rate (ΔE%). Simple linear regression was applied to examine the influence of various factors on the elastic modulus of nucleus pulposus cells. All statistical tests were two-tailed, and a *p* value of <0.05 was considered statistically significant.

## 3. Results

### 3.1. Clinical Characteristics and Radiographic Assessment of the Study Population

A total of 18 IVD specimens were harvested from patients undergoing ACDF. Degeneration severity was evaluated based on preoperative T2-weighted MRI and the Miyazaki grading system. Accordingly, subjects were stratified into three cohorts: the Mild group (*n* = 3), the Moderate group (*n* = 5), and the Severe group (*n* = 10).

Specimen distribution across spinal levels was uniform, comprising C3–4 (*n* = 6), C4–5 (*n* = 6), and C5–6 (*n* = 6). Age distribution was categorized into decadal subgroups: 50–59 years (*n* = 9) and 60–69 years (*n* = 9). As presented in Table 2, statistical analysis demonstrated distinct homogeneity in baseline characteristics across the three groups.

Regarding demographic characteristics, Chi-square analysis indicated no statistically significant differences in sex composition (*p* = 0.627), age distribution (*p* = 0.766), or affected spinal level (*p* = 0.148) among the mild, moderate, and severe groups.

To further preclude confounding factors in physical and temporal dimensions, Body Mass Index (BMI) and disease duration were analyzed. One-way analysis of variance (ANOVA) revealed that patient BMI remained at a uniform level across the three groups (20.35~21.44 kg/m^2^, *p* = 0.855), effectively ruling out potential interference from variations in weight-bearing load on the physical state of the disc. Concurrently, the disease duration (duration of symptoms) was highly consistent across groups (approximately 8 months, *p* = 0.981), indicating that inter-group differences did not stem from the cumulative temporal effects of disuse atrophy.

In summary, statistical results confirm high consistency across the three cohorts in terms of demographics, physical load, and disease duration. This ensures that the subsequently observed variations in cellular biomechanics are primarily attributable to the pathological status of disc degeneration.

### 3.2. Radiographic Quantification of Degeneration and Structural Compromise

To corroborate the validity of the stratification strategy at the macroscopic structural level, disc height loss and segmental ROM were quantified for all subjects. Detailed data are consolidated in Table 3.

Since the disc height loss score is an ordinal variable, the non-parametric Kruskal–Wallis test was employed, revealing a significant increasing trend concomitant with the progression of Miyazaki grades (Kruskal–Wallis statistic = 7.118, *p* = 0.0199). Specifically, Dunn’s multiple comparisons test indicated that while the height loss score in the Severe group (2.700 ± 0) was numerically higher than that of the Mild group (1.667 ± 0.471), this pairwise comparison showed a strong trend but did not reach statistical significance (adjusted *p* = 0.0988), likely due to the conservative nature of the non-parametric test and the limited sample size in the Mild cohort. Nevertheless, the overall significant variance confirms that severe degeneration is associated with substantial structural collapse.

Concurrently, a highly significant inverse correlation was observed between ROM and degeneration severity (W = 34.22, *p* = 0.0008). The Mild group preserved considerable mobility (13.667° ± 1.247°), whereas the Severe group exhibited a marked reduction to 4.700° ± 1.886° (*p* = 0.0031 vs. Mild group, Games-Howell test), indicating pathological macroscopic stiffness in severely degenerated segments.

These findings substantiate the stratification rationale employed in this study, characterizing the Severe group as a state of profound structural collapse and extreme stiffness. This macroscopic physical locking establishes a critical pathological context for the biomechanical alterations subsequently identified at the microscopic cellular level.

### 3.3. Determinants of Nucleus Pulposus Cell Elastic Modulus and Degeneration Patterns

To systematically investigate the variations in cellular biomechanics during IVD degeneration and identify potential influencing factors, NPCs were subjected to high precision nanomechanical scanning using AFM. Detailed statistical data are consolidated in Table 4.

**Figure 2:**
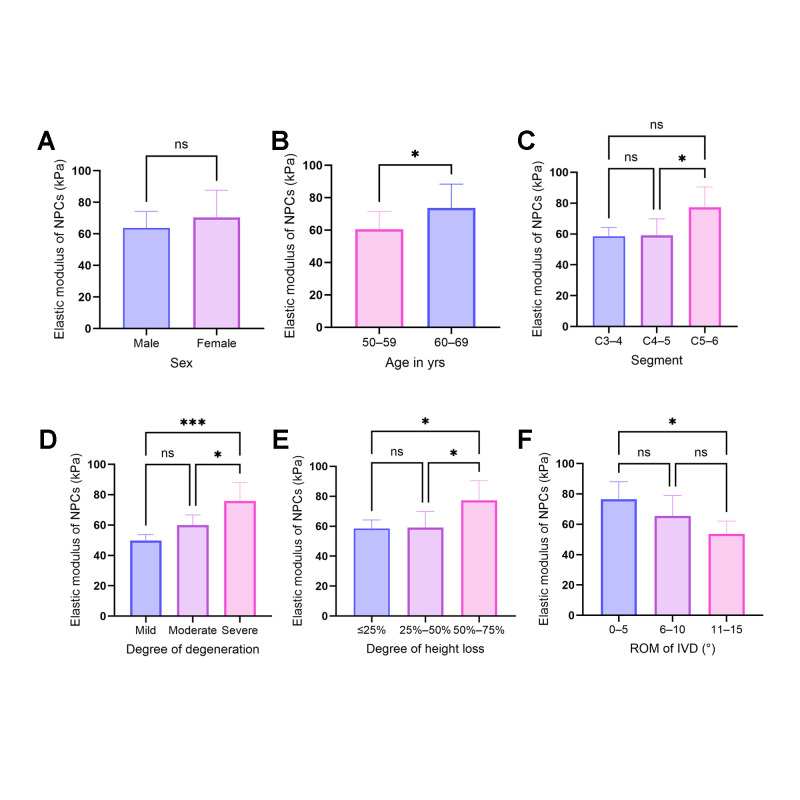
Impact of Demographic, Segmental, and Pathological Factors on the Baseline Elastic Modulus of Nucleus Pulposus Cells. (**A**–**C**) Influence of baseline biological variables on cellular biomechanics: (**A**) Cell stiffness showed no statistically significant difference between male and female subjects (ns). (**B**) Young’s modulus of cells increased significantly with advancing age, showing elevated stiffness in the older cohort (60–69 years). (**C**) Cells from different cervical segments exhibited comparable baseline stiffness, with no statistically significant craniocaudal gradient observed (ns). (**D**–**F**) Impact of pathological degeneration severity and macroscopic physical status: (**D**) Cell stiffness exhibited a significant increase concomitant with higher Miyazaki degeneration grades. The Severe group was significantly stiffer than both the Mild and Moderate groups. (**E**) Cell stiffness was significantly elevated in the severe collapse group (disc height loss 50–75%) compared to the moderate collapsed groups. (**F**) The extreme stiffness group (ROM 0–5°) displayed the highest Young’s modulus, significantly exceeding that of the most mobile group (11–15°). These findings confirm the concordance between macroscopic joint stiffness and microscopic cellular stiffening. Data are presented as mean ± SD. *** *p* < 0.001, * *p* < 0.05, ns: not significant.

Initial analysis revealed the specific influences of demographic and spinal segmental factors on the baseline elastic modulus of NPCs (Figure 2A–C). Regarding sex-dependent differences, although the mean elastic modulus in female patients (70.390 ± 16.299 kPa) appeared higher than in the male cohort (63.821 ± 9.864 kPa), an independent samples *t*-test with Welch’s correction revealed no statistically significant difference (t = 0.975, *p* = 0.3470). Concurrently, cellular biomechanics exhibited distinct age-dependency; cell stiffness in the older cohort (60–69 years; 73.667 ± 13.859 kPa) was significantly elevated compared to the younger cohort (50–59 years; 60.543 ± 10.313 kPa) (t = 2.149, *p* = 0.0487). This confirms that the aging process is intrinsically accompanied by mechanical remodeling at the cellular level. Furthermore, while cell stiffness across different cervical levels displayed a craniocaudal increasing trend, peaking at the C5–6 segment (73.918 ± 12.603 kPa) compared to C3–4 (61.369 ± 12.904 kPa) and C4–5 (66.028 ± 13.076 kPa), this variation did not reach statistical significance in the current sample size (F = 1.216, *p* = 0.3240).

Subsequent correlation analysis between cellular biomechanics and clinical macroscopic pathological parameters revealed a high degree of concordance between elastic modulus and degeneration severity, structural collapse, and limited mobility (Figure 2D–F). As MRI (Miyazaki) degeneration grades increased, cell stiffness demonstrated a significant stepwise upward trend (W = 16.15, *p* = 0.0013), escalating from 49.861 ± 3.202 kPa in the Mild group to 75.863 ± 11.748 kPa in the Severe group. Structural physical collapse also exerted a profound impact on cellular mechanics (W = 5.579, *p* = 0.0281). Notably, the severe collapse group (height loss > 50–≤75%) presented an elastic modulus of 77.255 ± 12.383 kPa, significantly higher than groups with mild or moderate loss. Finally, analysis of segmental range of motion (ROM) indicated intrinsic consistency between macroscopic joint stiffness and microscopic cellular stiffening (W = 6.526, *p* = 0.0169). Patients presenting clinically with extreme stiffness (ROM 0–5°) exhibited the highest cell stiffness (76.419 ± 10.781 kPa), whereas cohorts preserving good mobility (11–15°) retained relatively compliant cells (53.727 ± 7.248 kPa).

### 3.4. CTS Significantly Reduces NPC Elastic Modulus Across All Groups

To evaluate the direct physical de-stiffening efficacy and universality of mechanical therapy, the elastic moduli of NPCs were reassessed following 24 h of 5% CTS stimulation. Detailed data are presented in Table 5.

**Figure 3:**
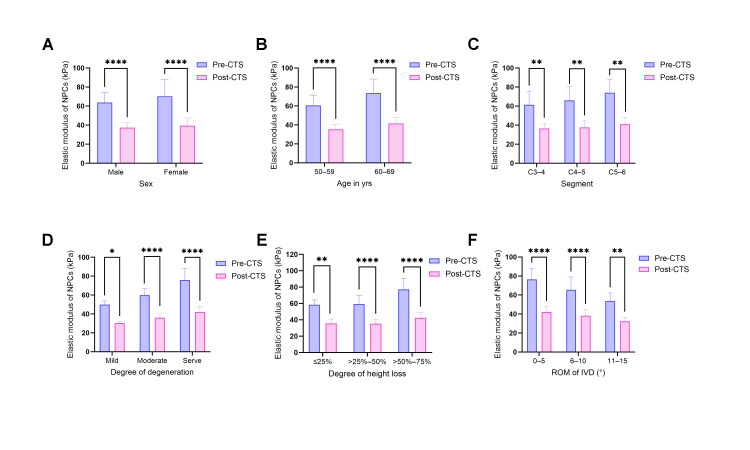
Universal De-stiffening Effect of Cyclic Tensile Strain (CTS) on Nucleus Pulposus Cell Elastic Modulus Across Diverse Subgroups. This figure illustrates the comparison of Young’s modulus between the static baseline (Pre-CTS, blue bars) and the post-intervention state (Post-CTS, red bars) stratified by various experimental conditions. (**A**–**C**) Influence of baseline demographic and segmental characteristics on therapeutic response: (**A**) Sex: Both male and female cells exhibited a significant reduction in elastic modulus following CTS intervention. (**B**) Age: Mechanical stimulation effectively reversed cell stiffness in both the younger (50–59 years) and older (60–69 years) cohorts. (**C**) Spinal Segment: Cells derived from different spinal levels (C3–4, C4–5, C5–6) demonstrated a consistent softening response to CTS. These data indicate that the therapeutic efficacy of CTS is universal and independent of the patient’s baseline biological background. (**D**–**F**) Influence of pathological degeneration status on therapeutic response: (**D**) Miyazaki Degeneration Grade: While baseline cell stiffness increased with degeneration severity, CTS significantly reduced stiffness across all grades (Mild, Moderate, and Severe). (**E**) Intervertebral Disc Collapse: CTS induced significant softening across groups stratified by varying degrees of disc height loss. (**F**) Segmental Range of Motion (ROM): Even in the “extreme stiffness group” (0–5°) with the most severe mobility restriction, CTS successfully restored cell stiffness to lower levels, with significant softening also observed in more mobile segments. Data are presented as mean ± SD. **** *p* < 0.0001, ** *p* < 0.01, * *p* < 0.05 compared to the corresponding Pre-CTS baseline.

AFM analysis confirmed that the modulation of cellular mechanics by physiological stretch is highly universal and independent of baseline biological characteristics (Figure 3A–C). A significant reduction in elastic modulus was observed post-intervention regardless of sex (*p* < 0.0001). Similarly, NPCs from different age strata (50–59 and 60–69 years; *p* < 0.0001) and spinal segments (C3–4, C4–5, and C5–6; *p* < 0.01) exhibited a consistent softening trend, with Young’s modulus values in all experimental groups significantly lower than their respective baseline (pre-CTS) measurements (Figure 3). This suggests that the cellular remodeling mechanisms triggered by mechanical stimulation are conserved and stable across diverse physiological backgrounds.

Further subgroup analysis stratified by pathological status revealed that CTS intervention exerts a significant “rescue” softening effect on cells exhibiting severe pathological alterations (Figure 3D–F). Regarding Miyazaki grading, the de-stiffening effect was observed in the Mild group (*p* = 0.0115) and Moderate group (*p* < 0.0001), but was most pronounced in the Severe group, where the elastic modulus plummeted from 75.863 ± 11.748 kPa to 42.026 ± 5.745 kPa (*p* < 0.0001), numerically approaching the baseline values of the Mild group. Parallel trends were observed in disc collapse and ROM classifications: in the severe collapse group (height loss > 50%) and the most restricted stiffness group (ROM 0–5°), Young’s moduli decreased to 42.359 ± 6.222 kPa (*p* < 0.0001) and 42.025 ± 5.401 kPa (*p* < 0.0001), respectively, following mechanical stimulation.

In summary, regardless of baseline characteristics or pathological status (degeneration, collapse, or stiffness), CTS significantly reduced the Young’s modulus of NPCs (*p* < 0.05). These findings provide compelling evidence for the universal and potent cellular de-stiffening efficacy of physical tensile strain.

### 3.5. The Degeneration Paradox: Maximal Mechanical Recovery Observed in Severe Degeneration, Collapse, and Specific Spinal Segments

**Figure 4:**
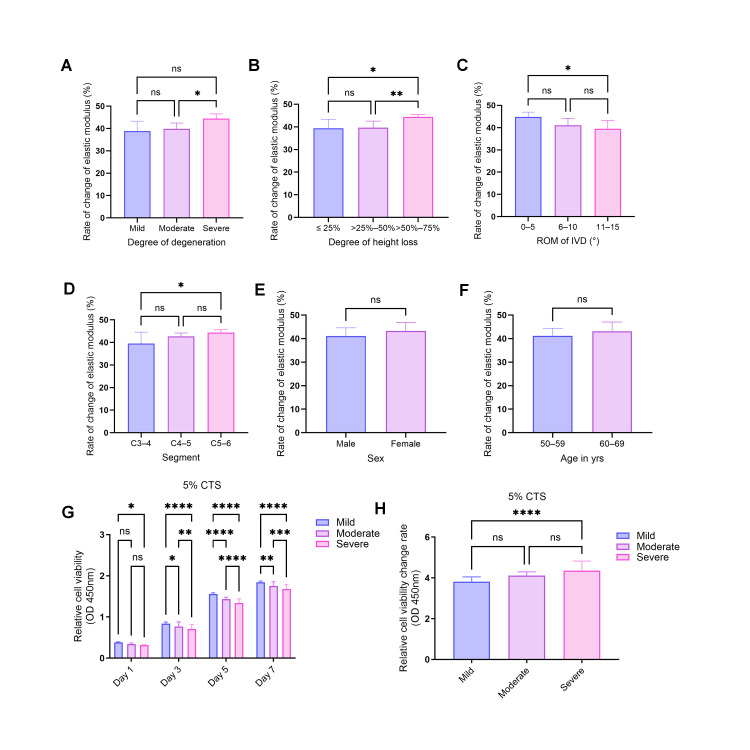
The “Degeneration Paradox” and Demographic Independence of Mechanical Recovery Rate (ΔE%) in Nucleus Pulposus Cells. This figure illustrates the relative rate of change in elastic modulus (ΔE%) across experimental groups following cyclic tensile strain (CTS) intervention. (**A**–**D**) Local pathological and anatomical factors exhibit a distinct “Degeneration Paradox,” indicating that greater pathological severity corresponds to higher physical recovery potential: (**A**) Miyazaki Grade: The recovery rate in the Severe group was significantly higher than in the Moderate groups. (**B**) Intervertebral Disc Collapse: The severe collapse group (height loss > 50%) displayed the highest ΔE%, significantly exceeding the moderate collapsed groups. (**C**) Segmental Range of Motion (ROM): Cells from the extreme stiffness group (0–5°) were most sensitive to mechanical stimulation, exhibiting the greatest magnitude of softening. (**D**) Segmental Distribution: Cells from the high-stress C5–6 segment exhibited a significantly higher recovery rate compared to the C3–4 segment. (**E**,**F**) Systemic demographic factors demonstrate “Independence”: No statistically significant differences in ΔE% were observed between (**E**) Sex and (**F**) Age subgroups (ns), confirming that mechanical recovery potential is not governed by systemic demographic variables. (**G**,**H**) CCK-8 analysis of cell proliferation dynamics under 5% CTS: (**G**) Absolute Viability (OD 450 nm): Assessed at Days 1, 3, 5, and 7. Results show a clear pathological hierarchy in absolute proliferation: Mild > Moderate > Severe. (**H**) Relative Cell Viability Change Rate: In contrast to absolute values, the Severe group exhibited the highest relative proliferative response to mechanical stimulation, significantly exceeding the Mild group. This biological “rebound” mirrors the mechanical recovery trends. Data are presented as mean ± SD. **** *p* < 0.0001, *** *p* < 0.001, ** *p* < 0.01, * *p* < 0.05, ns: not significant.

Although CTS universally induced cellular softening across all cohorts, further analysis of the relative change rate in elastic modulus (ΔE%) revealed a distinct “Degeneration Paradox” (Figure 4). Detailed data are presented in Table 6.

The results indicate that the potential for physical recovery is not only positively correlated with the severity of tissue pathology but also exhibits marked anatomical segmental specificity. Notably, when comparing distinct pathological stages, the recovery rate in the Severe group reached 44.420 ± 2.049%, significantly surpassing that of the Moderate group (39.797 ± 2.366%; Games-Howell adjusted *p* = 0.0289; Figure 4A). While the global variance across all three Miyazaki grades (Welch’s ANOVA) exhibited a strong but marginally significant trend (W = 6.308, *p* = 0.0516), this is mathematically attributable to the restricted sample size of the Mild cohort (*n* = 3), which diluted the omnibus statistical power. Indeed, the pairwise comparison between the Severe and Mild (38.846 ± 3.627%) groups did not reach statistical significance (adjusted *p* = 0.2727) due to this identical sample size limitation, yet the core trajectory of the “Degeneration Paradox” remains structurally intact.

Parallel significant trends were observed regarding the extent of disc collapse (W = 8.849, *p* = 0.0297; Figure 4B) and segmental range of motion (W = 5.144, *p* = 0.0418; Figure 4C). Specifically, the severe collapse group (height loss > 50%) achieved a mechanical recovery rate of 45.013 ± 1.782%. Consistent with the degeneration grade findings, this recovery rate was significantly higher than that of the moderate collapse group (*p* = 0.0108), whereas the comparison with the mild collapse group remained non-significant (*p* = 0.2738). Similarly, while the overall variance across ROM subgroups was statistically significant, the extreme stiffness group (ROM 0–5°) achieved the highest nominal mechanical recovery rate (44.874 ± 1.966%). Although its pairwise comparison with the restricted mobility group (6–10°) demonstrated a robust trend (*p* = 0.0579), it did not cross the strict significance threshold of the Games-Howell correction. Furthermore, cells from distinct spinal levels displayed highly significant variations in recovery (F = 3.931, *p* = 0.0453; Figure 4D). The C5–6 segment, which typically sustains the highest stress concentration, exhibited the maximal recovery rate (44.412 ± 1.076%), significantly exceeding that of the C3–4 segment (*p* = 0.0390).

Collectively, these findings imply that cells situated within environments characterized by severe degeneration, high mechanical stress, and extreme stiffness possess internal high-tension structures that are most sensitive to physical mobilization signals. Consequently, these cells undergo the most drastic mechanical remodeling when subjected to dynamic tensile strain.

To ascertain whether this heightened recovery rate is influenced by intrinsic biological characteristics, further analysis revealed that ΔE% exhibits demographic independence. No statistically significant differences were observed between males and females (43.306% vs. 41.107%, *p* = 0.2022; Figure 4E) or between the 50–59 and 60–69 age cohorts (41.248% vs. 43.166%, *p* = 0.2696; Figure 4F). This provides compelling evidence that mechanical recovery potential is primarily determined by the local microenvironmental status of the cell (pathological degeneration, structural collapse, and anatomical properties) rather than the patient’s systemic demographic characteristics.

To further validate whether this micromechanical “de-stiffening” translates into tangible biological functional improvements, cell proliferation dynamics were evaluated using the CCK-8 assay (Figure 4G,H). Under sustained 5% CTS stimulation, the absolute proliferative capacity maintained a distinct pathological hierarchy (Mild > Moderate > Severe), aligning with the axiom that degeneration precipitates a decline in basal vitality. However, a diametrically opposed trend emerged regarding the relative viability change rate. The Severe group demonstrated the most robust relative proliferative response, with a proliferation fold-change significantly higher than that of the Mild group (*p* < 0.0001). This biological resurgence mirrors the mechanical recovery trends, confirming that while severely degenerated cells possess the most compromised absolute baseline, they derive the maximal marginal benefit from mechanical therapy.

### 3.6. Mechanism: CTS Induces Cell Softening via F-Actin Depolymerization

**Figure 5:**
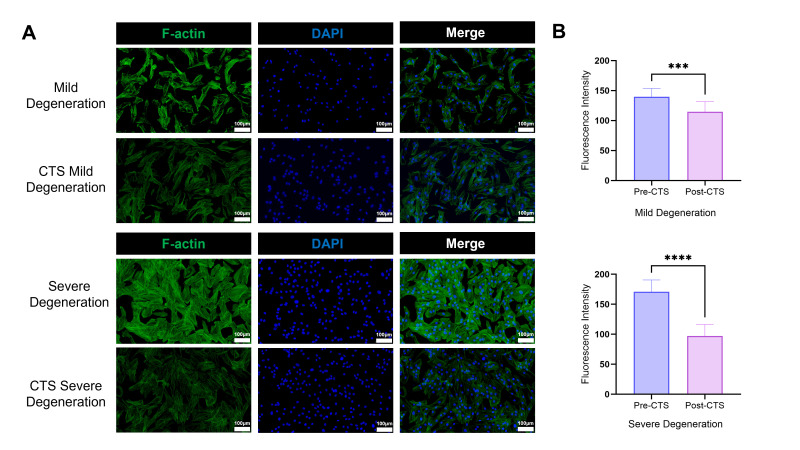
Immunofluorescence Analysis of Cyclic Tensile Strain (CTS)-Induced F-actin Cytoskeletal Depolymerization. This figure illustrates the cytoskeletal remodeling of F-actin in cells from Mild and Severe degeneration groups under static baseline (Pre-CTS) and CTS intervention conditions. (**A**) Representative immunofluorescence images: F-actin filaments were visualized using FITC-phalloidin staining (green), and nuclei were counterstained with DAPI (blue). Baseline (Pre-CTS): At baseline, the Severe group exhibited a significantly thicker and denser network of F-actin stress fibers compared to the Mild group, forming a characteristic “rigid cage-like” architecture. CTS Group: Following 24 h of intervention, F-actin depolymerization was observed in both groups. Notably, images of the Severe + CTS group reveal that the originally dense fiber bundles completely disintegrated into diffuse punctate structures, accompanied by a marked reduction in fluorescence intensity. (**B**) Quantitative analysis of Mean Fluorescence Intensity (MFI): The bar graph quantifies variations in F-actin fluorescence intensity. Results confirm that CTS induced a significant decline in MFI across both groups. Crucially, the magnitude of reduction in the Severe group was significantly greater than in the Mild group, corroborating the high mechanical recovery rates detected via AFM. Scale bar = 100 μm. **** *p* < 0.0001; *** *p* < 0.01.

To elucidate the subcellular structural basis underlying the observed physical de-stiffening (Section 3.4) and the heightened mechanical recovery rate in the severe degeneration group (Section 3.5), the remodeling patterns of the F-actin cytoskeleton were analyzed using fluorescence microscopy before and after 5% CTS intervention. A specific focus was placed on contrasting the Mild and Severe degeneration groups (Figure 5).

At baseline (Pre-CTS), NPCs with varying degrees of degeneration exhibited distinct cytoskeletal architectures. F-actin fibers in the Mild group appeared relatively sparse and thin. In sharp contrast, the cytoplasm of cells in the Severe group was populated by thick, densely packed, and interwoven stress fibers, forming a characteristic “rigid cage-like” structure. This significant morphological disparity correlates strongly with the high baseline stiffness (75.8 kPa) measured by AFM in the Severe group, corroborating that the dense F-actin network serves as the primary structural source of cellular stiffness.

Following 24 h of physiological CTS stimulation, imaging revealed extensive depolymerization of the F-actin network. Compared to the mild changes observed in the Mild group characterized by fiber thinning and a moderate reduction in fluorescence intensity cells in the Severe group underwent more drastic structural disintegration. The originally dense stress fiber bundles fragmented and vanished, transforming into diffuse punctate or short rod-like structures. Concurrently, the MFI of F-actin exhibited a precipitous decline.

This profound morphological contrast from extremely dense to widely diffuse provides a direct cytoskeletal structural explanation for the high ΔE% observed in the Severe group via AFM. These findings demonstrate that the macroscopic softening of severely degenerated cells results from mechanical stimulation–induced depolymerization and relaxation of the rigid F-actin cytoskeleton.

### 3.7. Correlation Analysis Between Elastic Modulus Recovery Rate and Clinical Parameters

**Figure 6:**
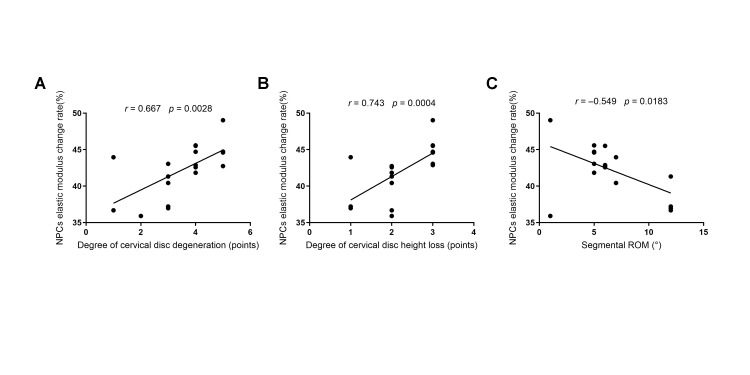
Scatter Plots Correlating Nucleus Pulposus Cell Mechanical Recovery Rate (ΔE%) with Clinical Pathological Parameters. This figure utilizes linear regression analysis to elucidate the relationship between microscopic cellular biomechanics and macroscopic clinical indices. (**A**) Miyazaki Degeneration Grade (*X*-axis) vs. ΔE% (*Y*-axis): A significant positive correlation (*r* = 0.667) is observed, indicating that increased radiographic degeneration severity is associated with a higher cellular recovery rate following mechanical intervention. (**B**) Intervertebral Disc Collapse (*X*-axis) vs. ΔE% (*Y*-axis): A significant positive correlation (*r* = 0.743) demonstrates that exacerbated structural collapse is accompanied by enhanced cellular mechanosensitivity. (**C**) Segmental Range of Motion (ROM) (*X*-axis) vs. ΔE% (*Y*-axis): A significant inverse correlation (r = −0.549) reveals the paradoxical relationship between macroscopic articular stiffness and the high latent recovery potential of cells at the microscopic level. The solid line represents the linear regression fit. *r* denotes the Spearman’s rank correlation coefficient in (**A**,**B**), and the Pearson correlation coefficient in (**C**). *p* < 0.05 indicates statistical significance.

To quantitatively elucidate the intrinsic relationship between clinical pathological parameters and the micromechanical recovery potential (ΔE%) of NPCs, Spearman’s rank correlation analyses (for ordinal variables) and Pearson correlation analyses (for continuous variables) were conducted, and linear regression scatter plots were generated (Figure 6).

The results provided dual statistical and visual confirmation of a significant linear dependence between cellular mechanical recovery rates and the patient’s macroscopic pathophysiological status.

First, a significant positive correlation was identified between the Miyazaki degeneration grade and the rate of change in NPC elastic modulus (Spearman r = 0.667, *p* = 0.0028). As degeneration severity increased, the distribution of data points along the *Y*-axis exhibited a distinct upward trajectory, lending strong support to the hypothesis that “greater degeneration corresponds to greater plasticity reserves” (Figure 6A).

Second, the degree of intervertebral disc collapse demonstrated a similarly significant positive correlation with the rate of modulus change (Spearman r = 0.743, *p* = 0.0004). This indicates that in patients exhibiting more pronounced structural collapse, the cellular physical recovery response at the microscopic level is proportionally more intense (Figure 6B).

Most notably, segmental range of motion (ROM) displayed a significant inverse correlation with the rate of change in cellular elastic modulus (Pearson r = −0.549, *p* = 0.0183). The negative slope of the regression line intuitively illustrates that greater macroscopic stiffness of the cervical spine (lower ROM) is associated with a paradoxically higher recovery rate in the constituent cells following CTS stimulation (Figure 6C).

This finding establishes a direct link between macroscopic stiffness and microscopic plasticity. It suggests that severely degenerated segments, which appear clinically rigid, actually harbor substantial latent physical recovery potential within their cellular architecture potential that awaits “unlocking” via appropriate mechanical stimulation such as CTS.

## 4. Discussion

By integrating AFM nanoindentation with refined clinical radiographic grading, the present study unveils the unique, non-linear mechanical response characteristics of severely degenerated human cervical NPCs under physiological mechanical stimulation. The experimental data rigorously challenge the traditional dogma that late-stage degenerated cells are in an irreversible terminal state of metabolic inertia. Instead, our findings substantiate a “physical unlocking” mechanism based on F-actin cytoskeletal remodeling. These insights not only deepen our understanding of intervertebral disc degeneration (IVDD) but also provide a cellular biomechanical theoretical basis for the application of mechanotherapy in advanced cervical spondylosis.

While previous research on IVDD has predominantly focused on biochemical changes, such as the upregulation of inflammatory factors and the activation of matrix degrading enzymes. However, mounting evidence suggests that the microenvironment surrounding cells significantly influences cell function through biophysical parameters [31,32,33]. This study underscores the central role of the physical dimension in pathological progression and elucidates the cross-scale mechanotransduction chain linking macroscopic tissue to microscopic cells. Clinically observed intervertebral space collapse and reduced segmental range of motion (ROM) are not merely anatomical changes; they map directly to an elevation in the Young’s modulus of the cell body at the microscopic level. This high concordance between macroscopic phenotype and microscopic mechanics is not coincidental but represents a pathological mechanoadaptive mechanism initiated by cells to cope with a deteriorating physical microenvironment. As posited by Edwin et al., cells utilize the cytoskeleton to maintain mechanical equilibrium between the intracellular and extracellular compartments [34]. NPCs are embedded within a fibrotic, dehydrated, and significantly stiffened ECM, they are likely compelled to hyper-assemble the F-actin cytoskeleton, potentially via the RhoA/ROCK signaling pathway, to maintain tensional homeostasis. This is hypothesized to generate high levels of internal actomyosin contractility to resist external matrix stiffness, a phenomenon consistent with the “feedback-driven increase in cell stiffness induced by a pathological mechanical microenvironment” described by Guo et al. [35]. Our AFM data reveal that cell stiffness in the Severe group reached as high as 75 kPa, indicating that cells may construct a rigid “protective shell” to compensate for environmental deterioration. However, this simultaneously appears to trap the cells in a physically locked state characterized by high rigidity and metabolic quiescence. Thus, the clinical manifestation of vertebral stiffness could essentially reflect the macroscopic accumulation of a vast number of cells in this “physically locked” state. Furthermore, while a numerical craniocaudal trend in cell stiffness was observed (peaking at the C5-6 segment), we must emphasize that macroscopic biomechanical loading is merely one likely contributor. This trend is undoubtedly influenced by a complex interplay of co-existing factors, including local segmental instability, anatomical variations, and specific local degenerative patterns, which warrant exploration in larger cohorts.

The most innovative finding of this study is the aforementioned “Degeneration Paradox,” wherein cells with the highest baseline stiffness and most severe degeneration grade exhibited the highest elastic modulus recovery rate under 5% CTS. This phenomenon can be deeply analyzed through the lens of “Soft Glassy Rheology (SGR)” and cytoskeletal fluidization theory proposed by Fabry et al. [36]. Severely degenerated cells, due to excessive accumulation and dense cross-linking of intracellular F-actin, are hypothesized to exist in a high-tension, high-energy metastable state. This state is analogous to the “glassy state” in physics: while static stiffness is extremely high, the system stores immense elastic energy and sits on the brink of thermodynamic instability, rendering it highly sensitive to external physical perturbations. The 5% CTS may act as a transient energy input capable of overcoming the energy barrier maintaining this high-tension state. As suggested by Xavier Trepat et al., dynamic stretching can instantaneously rupture weak bonds between actin and cross-linking proteins (e.g., F-actinin), precipitating a phase transition of the cytoskeleton from a solid-like glassy state to a liquid-like fluid state [37]. In contrast, mildly degenerated cells likely exist in a low-tension steady state, where the cytoskeleton exhibits elastic recoil rather than structural reorganization. Consequently, due to their high potential energy and structural brittleness, severe cells are proposed to undergo drastic structural collapse and reconfiguration during physical relaxation, releasing the maximal rebound space. From a biophysical perspective, this provides a theoretical framework to explain why the more severe the pathology, the more significant the physical plasticity.

It is important to acknowledge that the remarkable relative recovery rate (ΔE%) observed in the Severe group partially reflects a mathematical “floor effect”. As our data indicate, regardless of their divergent initial stiffness, all cohorts converged toward a similar compliant baseline (approximately 30–42 kPa) following CTS-induced cytoskeletal fluidization. Consequently, the Severe cohort, starting from the highest initial baseline, inevitably exhibited the largest mathematical drop. However, rather than negating the concept of mechanoplasticity, this convergence precisely underscores its biological significance. It elegantly demonstrates that despite undergoing severe pathological stiffening and dense F-actin accumulation, these late-stage cells are not irreversibly solidified. They retain the profound structural capacity to undergo dynamic cytoskeletal depolymerization and return to a near-physiological tensional baseline when provided with appropriate physical unloading.

Further examining this paradox from a bioenergetics perspective, the high-tension state of severely degenerated cells essentially represents a high-energy-cost pathological adaptation. According to Kuo et al., maintaining sustained actomyosin contraction consumes substantial ATP [38], which exacerbates “metabolic starvation” in the already ischemic and hypoxic microenvironment of the disc. The CTS-induced cytoskeletal fluidization observed in this study may trigger a “Metabolic Reprogramming” mechanism. By physically forcing the depolymerization of energy-intensive stress fibers, cells can reallocate limited ATP resources from maintaining structural tension to the synthesis and secretion of matrix proteins. Therefore, the therapeutic effect of CTS may stem partially from reducing the “Metabolic Cost of Contractility” required to maintain homeostasis, thereby opening up survival space within the energy-deficient context of late-stage degeneration.

Delving into the microscopic mechanism, F-actin depolymerization constitutes the critical switch for reversing cellular metabolic silence. Unlike traditional pharmacological treatments that struggle to penetrate dense matrix barriers, CTS acts directly on the physical structure of the cell, serving as a mechanotransduction mediator to unlock cellular function [39]. Immunofluorescence analysis confirmed that following CTS intervention, the F-actin network transformed from dense bundles to diffuse punctate structures. This remodeling has profound biological significance. First, it may alleviate physical constraints on the cell nucleus. Research by Simon et al. demonstrates that the cytoskeleton is directly connected to the nucleus via the Linker of Nucleoskeleton and Cytoskeleton (LINC) complex [40]; therefore, we hypothesize that a rigid F-actin cage potentially restricts nuclear micro-deformation, which could lead to increased nuclear envelope tension, chromatin condensation, and gene silencing. Conversely, F-actin depolymerization could reduce nuclear envelope tension and promote chromatin opening, thereby helping to restart suppressed transcriptional programs. Second, this process is hypothesized to reconstruct mechanical signaling pathways [41,42,43]. Many mechanosensitive transcription factors (e.g., Yes-associated protein (YAP)/TAZ or MRTF-A) are typically anchored to the cytoskeleton or sequestered in the cytoplasm. When the F-actin structure undergoes fluidization and rearrangement, these factors may be released and translocate to the nucleus to activate anabolic genes such as Col II [44,45]. This suggests that prior to biochemical metabolic impairment, cells first undergo physical structural solidification; CTS, through mandatory physical stretching, assists cells in crossing the “physical checkpoint” hindering regeneration, enabling them to regain the capacity to sense the microenvironment and synthesize matrix.

It is noteworthy that this physical remodeling induced by CTS may possess a “Mechanical Memory” effect, as cellular adaptation to mechanical environments exhibits temporal hysteresis [46]. Although this study only observed acute cytoskeletal depolymerization, such transient F-actin softening may alter nuclear pore permeability and the methylation state of chromatin (e.g., H3K9me3) by reducing LINC complex traction on the nuclear envelope. This implies that 5% CTS may not be merely a one-time physical relaxation but could leave a “de-stiffening” imprint at the epigenetic level, allowing cells to maintain a metabolically active state for an extended period after the withdrawal of mechanical stimulation.

The aforementioned cellular biomechanical findings offer a novel perspective for clinical therapeutic strategies, suggesting that physical therapy should be redefined as a form of “cytomechanical medicine”. Clinically, there is a tendency to view conservative treatment as futile for patients with Miyazaki grade IV–V degeneration and severe mobility restriction, under the assumption that disc function is irretrievably lost. However, our multivariate regression analysis reveals that patients with the worst ROM and most severe degeneration possess the greatest potential for mechanical recovery at the cellular level. This implies that physical therapy should not be viewed solely as a symptomatic measure to relieve muscle spasm, but as an intervention capable of directly inducing cellular de-stiffening and reversing pathological phenotypes. Accordingly, we propose a “mechan-sensitization strategy” for severe degeneration: adopting a sequential treatment mode that first utilizes physical therapy to induce cytoskeletal fluidization and unlock the physical state—restoring cellular sensitivity to biochemical signals—followed by biological therapies to generate synergistic effects. As long as cells have not undergone total apoptosis, even if radiographic imaging suggests a terminal stage, their microstructure retains significant plasticity. Unlocking this state via physical means may be the key to awakening dormant cells and retarding the degenerative process.

However, the exploitation of this mechanoplasticity must strictly adhere to the principle of “Mechanohormesis”. Although this study confirms that severely degenerated cells exhibit superior resilience under 5% strain, given the physical characteristics of their high-tension cytoskeleton [47], they display extreme susceptibility to overloading. Previous studies indicate that the mechanical window for improving degenerated discs is narrow [17,48]. Therefore, precise amplitude control is critical for physical therapy in late-stage patients: insufficient strain fails to cross the “vitrification” energy barrier, while excessive strain (e.g., >15%) may cause irreversible rupture of the fragile cytoskeleton and subsequent anoikis.

Despite confirming the mechanical plasticity of degenerated cells, this study has limitations. First, a notable limitation is the relatively small and unbalanced sample size across the degeneration cohorts, particularly in the Mild group (*n* = 3). This pronounced imbalance inherently reflects the clinical reality of obtaining human surgical specimens, as patients with mild cervical disc degeneration rarely require surgical intervention. Given this limited sample size, the Mild cohort is statistically more susceptible to individual outliers, which could substantially shift the group mean and variance. To mitigate this risk and ensure the robustness of our multi-group comparisons, we employed Welch’s ANOVA followed by Games-Howell post hoc tests, which are less sensitive to unequal variances and unbalanced group sizes. Second, while the 2D planar, normoxic, type I collagen-based culture model eliminated matrix interference to purify the cellular response, it cannot fully replicate the *in vivo* 3D osmotic and hypoxic microenvironment; thus, the current results should be regarded as hypothesis-generating, requiring validation in 3D hydrogel/organoid or *ex vivo* disc culture systems that better reproduce NP mechanics. Third, our AFM protocol specifically targeted short spindle-shaped cells. While this morphology represents the predominant phenotype of P2 NPCs in sub-confluent 2D culture and this strict selection was methodologically essential to eliminate morphological confounding factors during paired pre/post-CTS comparisons, it remains a limitation. Given the vast phenotypic heterogeneity of degenerated NPCs *in vivo* (which includes rounded, chondrocyte-like, and highly fibrotic subpopulations), this selection may inherently bias our stiffness measurements toward a specific subpopulation and not fully capture the entire biomechanical spectrum. Fourth, this study focused on physical phenotypic observations (stiffness, cytoskeleton); the conceptual framework involving specific molecular regulatory networks (e.g., YAP nucleocytoplasmic shuttling or RhoA/ROCK signaling) is presented as a hypothesis-consistent interpretation rather than proven mechanistic fact, requiring elucidation via detailed molecular biology experiments. Additionally, while we primarily interpret the profound de-stiffening of severely degenerated cells post-CTS as a beneficial ‘unlocking’ of the cytoskeleton, alternative interpretations must be explicitly acknowledged. Given the inherently fragile and high-tension nature of the F-actin network in these late-stage cells, such a marked drop in Young’s modulus could simultaneously reflect partial cytoskeletal collapse due to mechanical fatigue, early apoptotic remodeling, or even sublethal mechanical damage that precedes anoikis. Although our CCK-8 data demonstrated a relative proliferative rebound—suggesting the cells did not undergo immediate, widespread death—this bulk metabolic assay cannot definitively distinguish reversible, beneficial biomechanical adaptation from mechanical injury at the single-cell level. Future studies incorporating high-resolution apoptosis tracking and single-cell sequencing are required to delineate the exact boundary between mechanoplasticity and mechanical damage. Finally, we only observed acute mechanical responses over 24 h, whether longer-term cyclic stretching (e.g., one week or one month) translates into durable histological regeneration and disc height recovery warrants support from animal studies and longitudinal clinical follow-ups.

## 5. Conclusions

In conclusion, this study establishes a definitive cross-scale mechanotransduction link in CIVDD, identifying cellular physical stiffening as a core pathological feature intrinsic to macroscopic structural collapse and segmental immobility. We provide compelling evidence for a “Degeneration Paradox,” wherein severely degenerated and stiffened NPCs possess a latent, high-energy metastable cytoskeleton that exhibits superior relative mechanoplasticity under physiological cyclic tensile strain. This physical “de-stiffening” is driven by the catastrophic depolymerization of F-actin stress fibers, which effectively unlocks the cell from a rigid, metabolically suppressed state. These findings fundamentally challenge the therapeutic nihilism surrounding late-stage “black disc” degeneration. Consequently, we propose a paradigm shift towards “Mechano-priming”—utilizing precise physical therapy to dismantle the biophysical barriers of cellular rigidity—as a crucial precursor to potentiate biological repair in severe cervical spondylosis.

## Figures and Tables

**Table 1:** Detailed clinical characteristics and mechanical properties of the 18 human cervical nucleus pulposus specimens.

IVD	Sex	Age (Years)	Segment	MRI Grade	Degree of Degeneration	Degree of Height Loss (Points)	ROM (°)	Elastic Modulus (kPa)	Post-CTS Elastic Modulus (kPa)	Rate of Change in Elastic Modulus
1	Female	54	C3–4	1	Mild	2	12	45.882 ± 16.271	29.055 ± 10.241	36.676%
2	Female	54	C4–5	3	Moderate	2	7	49.551 ± 16.437	29.517 ± 9.732	40.431%
3	Female	54	C5–6	4	Severe	3	5	56.915 ± 21.375	31.468 ± 11.752	44.709%
4	Female	68	C3–4	5	Severe	3	1	85.445 ± 13.856	43.553 ± 7.034	49.027%
5	Female	68	C4–5	5	Severe	3	5	89.081 ± 16.153	49.342 ± 8.915	44.609%
6	Female	68	C5–6	4	Severe	3	6	92.508 ± 29.608	50.402 ± 16.075	45.515%
7	Female	57	C3–4	3	Moderate	2	12	65.326 ± 10.246	38.332 ± 5.984	41.322%
8	Female	57	C4–5	5	Severe	2	6	69.320 ± 8.261	39.694 ± 4.709	42.738%
9	Female	57	C5–6	5	Severe	3	5	79.480 ± 26.852	43.926 ± 14.781	44.732%
10	Male	69	C3–4	3	Moderate	1	6	64.881 ± 22.823	40.741 ± 14.274	37.208%
11	Male	67	C4–5	1	Mild	1	15	53.723 ± 19.126	30.108 ± 10.652	43.956%
12	Male	67	C5–6	3	Moderate	3	8	63.214 ± 22.645	36.001 ± 12.838	43.051%
13	Male	51	C3–4	2	Mild	2	14	49.975 ± 12.551	32.0312 ± 8.004	35.906%
14	Male	51	C4–5	4	Severe	2	7	61.871 ± 16.680	35.991 ± 9.659	41.829%
15	Male	51	C5–6	4	Severe	3	5	66.571 ± 17.678	38.021 ± 10.054	42.886%
16	Male	69	C3–4	3	Moderate	1	10	56.703 ± 18.179	35.737 ± 11.405	36.975%
17	Male	69	C4–5	4	Severe	2	4	72.621 ± 6.507	41.698 ± 3.722	42.581%
18	Male	69	C5–6	4	Severe	3	3	84.821 ± 10.714	46.163 ± 5.811	45.576%

Note: ‘Elastic modulus’ represents the baseline (Pre-CTS) cell stiffness evaluated via Atomic Force Microscopy (AFM) nanoindentation. ‘Post-CTS Elastic modulus’ indicates the stiffness measured immediately following 24 h of 5% cyclic tensile strain intervention using the Flexcell system. The ‘Rate of change in elastic modulus’ (ΔE%) is calculated as (Baseline modulus − post-CTS modulus)/Baseline modulus × 100%. Detailed methodological protocols for the evaluation of these variables are comprehensively described in Methods Section 2.3 and Section 2.4.

**Table 2:** Demographic and baseline clinical characteristics of the study population.

NPCs		Degree of Degeneration	
	Mild Degeneration	Moderate Degeneration	Severe Degeneration
Total (*n* = 18)	3	5	10
Sex			
Male (*n* = 9)	2	3	4
Female (*n* = 9)	1	2	6
χ^2^ Value		0.933	
*p* Value		0.627	
Age in years			
50–59 (*n* = 9)	2	2	5
60–69 (*n* = 9)	1	3	5
χ^2^ Value		0.533	
*p* Value		0.766	
Segment			
C3–4 (*n* = 6)	2	3	1
C4–5 (*n* = 6)	1	2	3
C5–6 (*n* = 6)	0	0	6
χ^2^ Value		9.481	
*p* Value		0.148	
BMI (kg/m^2^)	20.351 ± 3.606	20.867 ± 3.415	21.442 ± 2.269
F Value		F (2, 15) = 0.1578	
*p* Value		0.855	
Disease duration (month)	8.000 ± 2.828	8.400 ± 2.939	8.400 ± 2.939
F Value		F (2, 15) = 0.01953	
*p* Value		0.981	

**Table 3:** Radiographic quantification of disc degeneration and physical collapse across study groups.

NPCs	Degree of Degeneration				
	Mild Degeneration	Moderate Degeneration	Severe Degeneration	Test Statistic	*p* Value
Total (*n* = 18)	3	5	10		
Degree of height loss (points)	1.667 ± 0.471	1.800 ± 0.471	2.700 ± 0	H = 7.118	0.0199
ROM (°)	13.667 ± 1.247	8.600 ± 2.624	4.700 ± 1.886	W = 34.22	0.0008

**Table 4:** Baseline elastic modulus of nucleus pulposus cells stratified by demographic, anatomical, and pathological factors.

IVDs	No. of Subjects	Elastic Modulus (kPa)
Total (*n* = 18)	18	67.105 ± 13.866
Sex		
Male (*n* = 9)	9	63.821 ± 9.864
Female (*n* = 9)	9	70.390 ± 16.299
T Value		t = 0.975 df = 13.17
*p* Value		0.3470
Age in years		
50–59 (*n* = 9)	9	60.543 ± 10.313
60–69 (*n* = 9)	9	73.667 ± 13.859
T Value		t = 2.149 df = 14.78
*p* Value		0.0487
Segment		
C3–4 (*n* = 6)	6	61.369 ± 12.904
C4–5 (*n* = 6)	6	66.028 ± 13.076
C5–6 (*n* = 6)	6	73.918 ± 12.603
F Value		F (2, 15) = 1.216
*p* Value		0.3240
Degree of height loss		
≤25%	3	58.436 ± 4.717
>25–≤50%	7	59.221 ± 9.876
>50–≤75%	8	77.255 ± 12.383
W Value		W (2, 8.573) = 5.579
*p* Value		0.0281
Degree of degeneration		
Mild degeneration	3	49.861 ± 3.202
Moderate degeneration	5	59.935 ± 6.043
Severe degeneration	10	75.863 ± 11.748
W Value		W (2, 8.542) = 16.15
*p* Value		0.0013
Range of motion (°)		
0–5 (*n* = 7)	7	76.419 ± 10.781
6–10 (*n* = 7)	7	65.436 ± 12.504
11–15 (*n* = 4)	4	53.727 ± 7.248
W Value		W (2, 9.323) = 6.526
*p* Value		0.0169

**Table 5:** Changes in elastic modulus of nucleus pulposus cells following 24-h cyclic tensile strain (CTS) intervention.

IVDs	No. of Subjects	Elastic Modulus (kPa)	Post-CTS Elastic Modulus (kPa)	*p* Value
Total (*n* = 18)	18	67.105 ± 13.866	38.432 ± 6.419	<0.0001
Sex				
Male (*n* = 9)	9	63.821 ± 9.864	37.387 ± 4.661	<0.0001
Female (*n* = 9)	9	70.390 ± 16.299	39.476 ± 7.648	<0.0001
Age in years				
50–59 (*n* = 9)	9	60.543 ± 10.313	35.337 ± 4.814	<0.0001
60–69 (*n* = 9)	9	73.667 ± 13.859	41.527 ± 6.329	<0.0001
Segment				
C3–4 (*n* = 6)	6	61.369 ± 12.904	36.574 ± 4.951	0.0039
C4–5 (*n* = 6)	6	66.028 ± 13.076	37.725 ± 6.868	0.0069
C5–6 (*n* = 6)	6	73.918 ± 12.603	40.997 ± 6.434	0.0015
Degree of height loss				
≤25%	3	58.436 ± 4.717	35.528 ± 4.343	0.0018
>25–50%	7	59.221 ± 9.876	35.188 ± 4.674	<0.0001
>50–75%	8	77.255 ± 12.383	42.359 ± 6.222	<0.0001
Degree of degeneration				
Mild degeneration	3	49.861 ± 3.202	30.398 ± 1.232	0.0115
Moderate degeneration	5	59.935 ± 6.043	36.065 ± 3.741	<0.0001
Severe degeneration	10	75.863 ± 11.748	42.026 ± 5.745	<0.0001
Range of motion (°)				
0–5 (*n* = 7)	7	76.419 ± 10.781	42.025 ± 5.401	<0.0001
6–10 (*n* = 7)	7	65.436 ± 12.504	38.298 ± 5.961	<0.0001
11–15 (*n* = 4)	4	53.727 ± 7.248	32.382 ± 3.597	0.0030

Note: ‘Post-CTS Elastic modulus (kPa)’ indicates the elastic modulus measured immediately after 24 h of 5% cyclic tensile strain stimulation.

**Table 6:** Rate of change in elastic modulus (ΔE%) stratified by demographic and clinicopathological factors.

IVDs	No. of Subjects	Rate of Change in Elastic Modulus
Total (*n* = 18)	18	42.207 ± 3.485
Sex		
Male (*n* = 9)	9	43.306 ± 3.329
Female (*n* = 9)	9	41.107 ± 3.283
T Value		t = 1.330, df = 16
*p* Value		0.2022
Age in years		
50–59 (*n* = 9)	9	41.248 ± 2.972
60–69 (*n* = 9)	9	43.166 ± 3.690
T Value		t = 1.145, df = 15.30
*p* Value		0.2696
Segment		
C3–4 (*n* = 6)	6	39.519 ± 4.594
C4–5 (*n* = 6)	6	42.691 ± 1.363
C5–6 (*n* = 6)	6	44.412 ± 1.076
F Value		F (2, 15) = 3.931
*p* Value		0.0453
Degree of height loss		
≤25%	3	39.379 ± 3.237
>25–≤50%	7	40.212 ± 2.589
>50–≤75%	8	45.013 ± 1.782
W Value		W (2, 4.317) = 8.849
*p* Value		0.0297
Degree of degeneration		
Mild degeneration	3	38.846 ± 3.627
Moderate degeneration	5	39.797 ± 2.366
Severe degeneration	10	44.420 ± 2.049 ^#^
W Value		W (2, 4.364) = 6.308
*p* Value		0.0516
Range of motion (°)		
0–5 (*n* = 7)	7	44.874 ± 1.966
6–10 (*n* = 7)	7	41.106 ± 2.906
11–15 (*n* = 4)	4	39.465 ± 3.319
W Value		W (2, 7.075) = 5.144
*p* Value		0.0418

Note: Data are presented as mean ± SD. Statistical comparisons were performed using Student’s *t*-test or Welch’s ANOVA. ^#^ adjusted *p* < 0.05 compared to the Moderate degeneration group (Games-Howell post hoc test).

## Data Availability

The original contributions presented in this study are included in the article. Further inquiries can be directed to the corresponding author.

## Supplementary Materials

The following supporting information can be downloaded at: https://www.mdpi.com/article/10.3390/biom16030461/s1, Figure S1: Representative T2-weighted midsagittal MRI scans illustrating the Miyazaki grading system for cervical intervertebral disc degeneration.

## Abbreviations

The following abbreviations are used in this manuscript:

ACDF	Anterior Cervical Discectomy and Fusion
AFM	Atomic Force Microscopy
ANOVA	Analysis of Variance
ATP	Adenosine Triphosphate
BMI	Body Mass Index
BSA	Bovine Serum Albumin
CCK-8	Cell Counting Kit-8
CIVDD	Cervical Intervertebral Disc Degeneration
Col II	Type II Collagen
CTS	Cyclic Tensile Strain
DAPI	4′,6-diamidino-2-phenylindole
DMEM/F-12	Dulbecco’s Modified Eagle Medium/Nutrient Mixture
ECM	Extracellular Matrix
F-actin	Filamentous actin
FBS	Fetal Bovine Serum
FITC	Fluorescein Isothiocyanate
IVD	Intervertebral Disc
IVDD	Intervertebral Disc Degeneration
LINC	Linker of Nucleoskeleton and Cytoskeleton
MFI	Mean Fluorescence Intensity
MMPs	Matrix Metalloproteinases
MRI	Magnetic Resonance Imaging
NP	Nucleus Pulposus
NPCs	Nucleus Pulposus Cells
OD	Optical Density
PBS	Phosphate-Buffered Saline
PFA	Paraformaldehyde
ROM	Range of Motion
ROI	Region of Interest
SD	Standard Deviation
SGR	Soft Glassy Rheology
YAP	Yes-associated protein
