# Supplementary material for: The Degeneration Paradox: Severely Degenerated Cervical Nucleus Pulposus Cells Display Enhanced Mechanoplasticity Under Moderate Cyclic Tensile Strain
Source: Biomolecules. 2026 Mar 18;16(3):461. doi: 10.3390/biom16030461 (PMC13024720; doi:10.3390/biom16030461)
Supplement: Supplementary file 1 [file biomolecules-16-00461-s001.zip › biomolecules-4175234-supplementary.pdf]

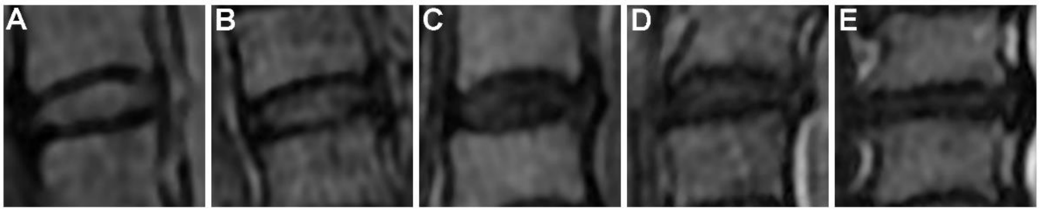

Figure S1. Representative T2-weighted midsagittal MRI scans illustrating the Miyazaki grading system for cervical intervertebral disc degeneration. The grading system evaluates four structural parameters: nucleus signal intensity, nucleus structure, distinction between the nucleus and annulus, and disc height.

(A) Grade I: Hyperintense, homogeneous white structure with clear distinction and normal disc height. (B) Grade II: Hyperintense, inhomogeneous structure containing a horizontal white band, with clear distinction and normal height. (C) Grade III: Intermediate signal intensity, inhomogeneous gray to black structure, unclear distinction, and normal to decreased height. (D) Grade IV: Hypointense, inhomogeneous gray to black structure, lost distinction, and normal to decreased height. (E) Grade V: Hypointense, inhomogeneous gray to black structure, completely lost distinction, and a collapsed disc space.

Note: Based on this specific radiographic evaluation, patients in the current study were stratified into the Mild (Grades I–II), Moderate (Grade III), and Severe (Grades IV–V) degeneration cohorts.
